# Disruption of three polyamine uptake transporter genes in rice by CRISPR/Cas9 gene editing confers tolerance to herbicide paraquat

**DOI:** 10.1007/s42994-022-00075-4

**Published:** 2022-06-25

**Authors:** Yu-Shu Lyu, Li-Miao Cao, Wen-Qian Huang, Jian-Xiang Liu, Hai-Ping Lu

**Affiliations:** 1 State Key Laboratory of Plant Physiology and Biochemistry, College of Life Sciences, Zhejiang University, Hangzhou, 310058 China; 2 State Key Laboratory of Rice Biology, Zhejiang Provincial Key Laboratory of Crop Genetic Resources, Institute of Crop Science, Zhejiang University, Hangzhou, 310058 China

**Keywords:** Paraquat, *OsPUTs*, CRISPR/Cas9, Rice

## Abstract

Weeds are a major biotic constraint that can cause dramatic crop production losses. Herbicide technology has been widely used by farmers as the most cost-effective weed control measure, and development of new strategy to improve herbicide tolerance in plants is urgently needed. The CRISPR/Cas9-based genome editing tool has been used in diverse applications related to agricultural technology for crop improvement. Here we identified three polyamine uptake transporter (*PUT*) genes in rice that are homologous to the *Arabidopsis* At*RMV1*. We successfully demonstrate that CRISPR/Cas9-targeted mutagenesis of *OsPUT1/2/3* greatly improves paraquat resistance in rice without obvious yield penalty. Therefore, manipulation of these loci could be valuable for producing transgene-free rice with improved herbicide resistance in future.

**Supplementary Information:**

The online version contains supplementary material available at 10.1007/s42994-022-00075-4.

## Introduction

Weeds have great impacts on crop yield. Herbicide technology has been increasingly used by farmers worldwide as the most cost-effective weed control measure. Paraquat (*N,N*′-dimethyl-4,4′-bipyridinium dichloride, also called methyl viologen), an effective and affordable non-selective herbicide, is one of the most widely used herbicides. It mainly targets chloroplasts by diverting electrons from photosystem I to oxygen, thus generating superoxide and hydrogen peroxide, which cause photooxidative stress (Bromilow 2003). Furthermore, paraquat is a close alternative to glyphosate, another broad-spectrum herbicide.

Although glyphosate is still widely used as a non-selective herbicide, glyphosate-resistance weeds are also developed (Kreiner et al. 2018). Paraquat would be a likely substitute, although paraquat is widely acknowledged to be more toxic than glyphosate and dangerous for animal and human being, resulting in a ban on paraquat use. However, the ban increased cropping production costs and caused large declines in farm profit (Walsh and Kingwell 2021). Therefore, optimization of paraquat use is urgent in crop production, such as developing paraquat-resistant crop germplasms.

Several paraquat-resistant genes were identified in *Arabidopsis,* such as the plasma membrane transport gene *AtPDR11* (pleiotropic drug resistance 11) (Xi et al. 2012) and the intracellular transport gene *AtPAR1* (paraquat-resistant 1) (Li et al. 2013). The dominant mutation in *AtDTX6* (detoxification efflux carrier 6) results in enhancing the substrate binding affinity and efflux activity of DTX6 (Lv et al. 2021; Xia et al. 2021). *AtPAR2* plays an important role in regulating cell death through modulating intracellular NO (nitric oxide) level when plants are treated with paraquat (Chen et al. 2009). Zhang et al. (2018) demonstrate that editing the uORF of *LsGGP2* (GDP-L-galactose phosphorylase 2) shows paraquat resistance in lettuce. However, to our knowledge, no paraquat resistance crops have yet been developed until now.

Previously, Fujita et al. (2012) reported that cell plasma membrane transporter AtRMV1 (resistant to methyl viologen 1, At5g05630) is responsible for uptake of polyamines and their analog paraquat in *Arabidopsis*. The *Arabidopsis rmv1* mutant exhibits paraquat tolerance, while the *RMV1* over-expression plants showed severe growth defects (Fujita et al. 2012). In the current study, we identified three *RMV1* homologous genes in rice and demonstrated the potential use of targeted mutagenesis of these three genes based on CRISPR/Cas9 technology for improving herbicide resistance in rice.

## Materials and methods

### Plant materials and growth conditions

*OsPUTs* knockout lines were generated by multiplex genome editing CRISPR (Ma et al. 2015) in a *japonica* variety Xidao #1 background. Rice seeds were germinated in water at 37 °C for 2 days. Germinated seeds were placed into 96-well plates and grown in Kimura B nutrient solution under white fluorescent light (20,000 lx, 16 h light/8 h dark) at 25 °C and with 65% relative humidity.

### Generation *OsPUTs*-KO rice plants by CRISPR/Cas9

The editing target in *OsPUT* was designed in CRISPR-GE program (http://skl.scau.edu.cn/home/) (Ma et al. 2015; Xie et al. 2017). The primers were listed in Table S1. After two-round PCR amplifications, a complete sgRNA expression cassette was formed. Three cassettes, namely OsU3-*OsPUT1* sgRNA, OsU6a-*OsPUT2* sgRNA, and OsU6b-*OsPUT3* sgRNA, were cloned into the pYLCRISPR/Cas9Pubi-H vector using the T4 ligation method. The ligated products were directly used to transform *E. coli* competent cells. The CRISPR/Cas9 constructs were introduced into *A. tumefaciens* strain EHA105 by electroporation and transgenic plants were generated via agrobacterial-mediated transformation. Genomic DNA extraction from leaves of transgenic rice plants was carried out using the CTAB (cetyltrimethylammonium bromide) method. The DNA fragment containing sgRNA target was amplified in the reaction containing 20 μL with 10 μL 2 × Taq mix buffer, 1 μL DNA and 0.5 μL 10 μM each primer. The primer used for *OsPUT1*: F: GTGGCTACATGCACATGGTTTG, R: AAGGGAGGAGGGAGAAGACG; For *OsPUT2*: F: GCAACCAACCAGCGACAGAGC, R: TGGAGAACCCGACGAGGAACG; For *OsPUT3*: F: GCTAGAATAATCTAGGACTCTTC, R: ACACCACTCAGCCACTTTGC. After 30 cycles (94 °C, 30 s; 58 °C, 30 s; 72 °C, 30 s), the PCR products were sequenced directly using forward primers. Homozygous mutants were selected and used in further experiment.

### Leaf floating test and TEM anaysis

The leaves detached from plants were floated in a buffer containing 3 mM MES [2-(N-morpholino) ethanesulfonic acid monohydrate] and 0.05% Tween-20, containing serious of concentrations of paraquat for 24 h under continuous light.

For Transmission Electron Microscope (TEM) analysis, the sample was first fixed with 2.5% glutaraldehyde in phosphate buffer (0.1 M, pH 7.0) for more than 4 h; washed three times in the phosphate buffer (0.1 M, pH 7.0) for 15 min at each step; then post-fixed with 1% OsO_4_ in phosphate buffer for 1–2 h and washed three times in the phosphate buffer for 15 min at each step. The sample was first dehydrated by a graded series of ethanol (30%, 50%, 70%, 80%, 90% and 95%) for about 15 min at each step, then dehydrated by alcohol for 20 min. At the end, transferred to absolute acetone for 20 min. The specimen was placed in 1:1 mixture of acetone and resin mixture for 1 h at room temperature, then transferred to 1:3 solution for 3 h. The specimen was sectioned in LEICA EM UC7, and observed in Hitachi Model H-7650 TEM.

### Measurement of paraquat by HPLC–MS

Leaf and root tissue powder was homogenized in 1 mL 50% methanol, followed by centrifugation at 12,000*g* for 10 min. The supernatant was used for subsequent HPLC–MS analysis. HPLC–MS analysis was performed using an Agilent 1260 HPLC device coupled with an Agilent 6460 triple quadrupole mass spectrometer (Agilent Technologies). A Syncronis HILIC (100 mm × 2.1 mm, 5 μm; ThermoFisher Scientific) column was used. Mobile phase A: 10 mM ammonium formate and 0.1% acetonitrile, and mobile phase B: acetonitrile. The samples were separated using gradient elution. The flow rate was 200 μL/min. The Agilent 6460 mass spectrometer was operated in the positive product ion scan mode.

## Results and discussion

**Fig. 1:**
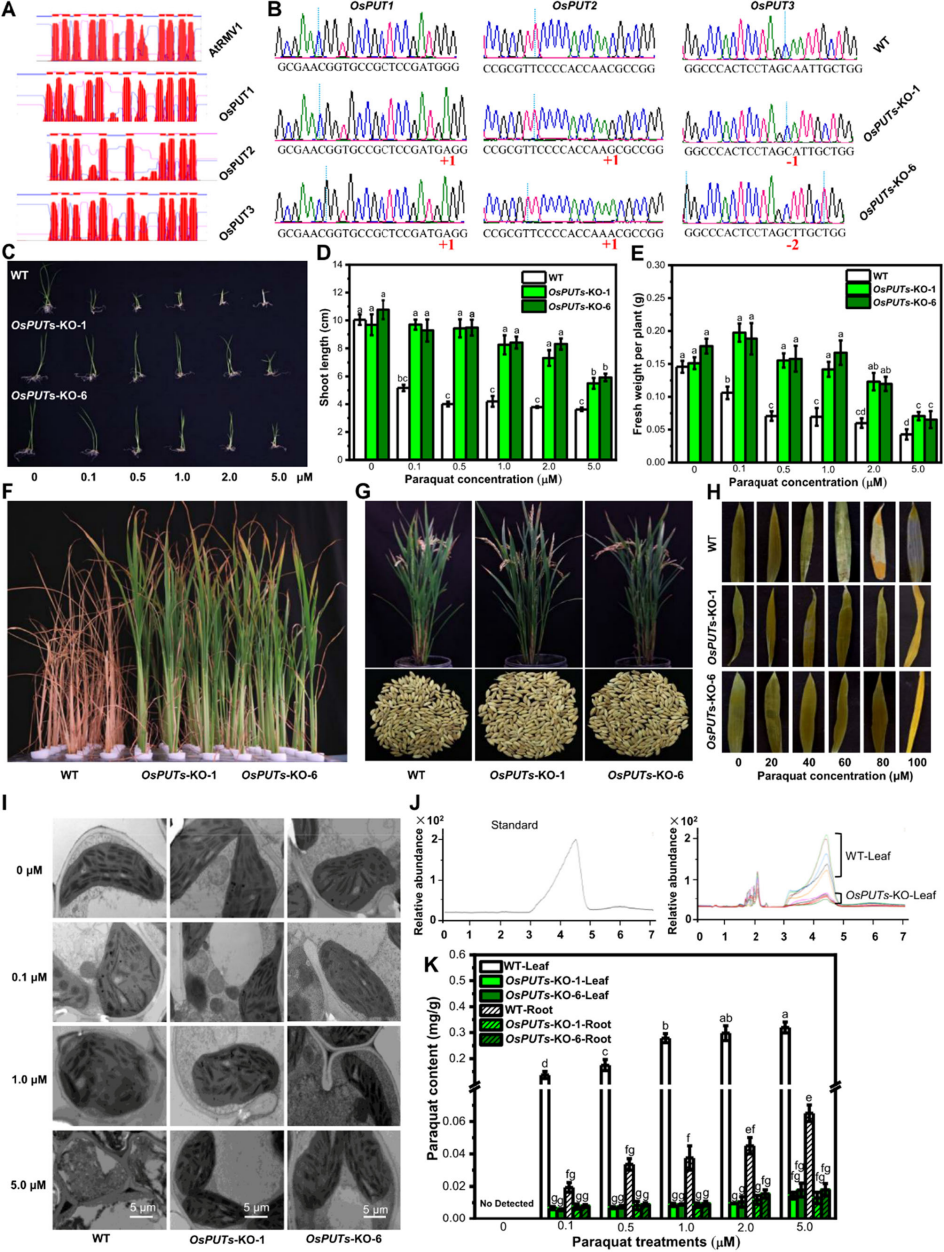
Gene editing of *OsPUT1/2/3* confers paraquat resistance in rice. **A** Predicted transmembrane structures of AtRMV1 and OsPUT1/2/3. **B** Mutations of *OsPUT1*/*2*/*3* in two independent triple mutant lines *OsPUTs-KO-1* and *OsPUTs-KO-6.* The insertion and deletion were marked with ± number, respectively. **C**–**E** Phenotypic analysis of paraquat resistance in one-week-old WT and *OsPUTs* mutant seedlings. **F**–**G** Phenotypic analysis of paraquat resistance in one-month-old WT and *OsPUTs* mutant plants sprayed with 200 μM paraquat. **H**–**I** Maintaining of chloroplast structural integrity in *OsPUTs* mutant plants after paraquat treatment. **J**–**K** Quantitative analysis of paraquat content by HPLC–MS in WT and *OsPUTs* mutant plants. Values are shown in mean ± s.e.m. (*n* = 10). Different letters denote significance (*P* < 0.05) in *t* test

To explore the possible applications of *AtRMV1*-like genes in rice (*Oryza Sativa*), three rice polyamine uptake transporter (PUT) genes were identified through BlastP, namely *OsPUT1* (Os02g0700500), *OsPUT2* (Os12g0580400) and *OsPUT3* (Os03g0576900). Similar transmembrane domains were predicted by TMHMM 2.0 among AtRMV1 and OsPUT1/2/3 (Fig. 1A). To know whether manipulation of *OsPUT1/2/3* enhances paraquat tolerance in rice, we generated *OsPUTs* mutant, with three target sites designed for *OsPUT1*/*2*/*3* by searching the CRISPR-GE program (http://skl.scau.edu.cn/home/) to minimize off-target effects (Ma et al. 2015; Xie et al. 2017). Twenty-four independent transgenic T_0_ plants were generated via *Agrobacterium tumefaciens*-mediated transformation of *japonica* rice genotype Xidao#1, and two independent lines of triple mutants, *OsPUTs*-KO-1 (an ‘A’ insertion in *OsPUT1*, a ‘G’ insertion in *OsPUT2*, and an ‘A’ deletion in *OsPUT3*) and *OsPUTs*-KO-6 (an ‘A’ insertion in *OsPUT1*, an ‘A’ insertion in *OsPUT2* and ‘AA’ deletion in *OsPUT3*) were identified and analyzed further (Fig. 1B). Off-target mutations are an important issue when considering functional gene analysis, as well as the molecular breeding of crop plants with large genome size. Three potential off-target sites (provide by CRISPR-GE program) were chosen for sequencing for each *OsPUT* gene, and no mutagenesis was detected in other sites (Table S2).

There were no morphological differences between the WT and these mutants under normal growth conditions (0 μM). However, after one-week treatment with paraquat, the wild type (WT) seedlings exhibited significant sensitivity even with a low concentration of paraquat (0.1 μM) (Fig. 1C). In contrast, both *OsPUTs*-KO-1 and *OsPUTs*-KO-6 seedlings were more resistant to paraquat than the WT plants, in terms of overall growth (Fig. 1C), shoot length (Fig. 1D) and plant weight (Fig. 1E). When treated with a relatively high concentration of paraquat (5.0 μM), both WT plants and mutants are sensitivity to paraquat. Furthermore, one-month old WT and *OsPUTs* triple mutant plants were treated with 200 μM paraquat in a foliar spray assay. About one week later, we observed that *OsPUTs* triple mutant plants grew normally with a little dry at leaf tip, but all WT plants started to dehydrate and eventually died (Fig. 1F). Meanwhile, no significant negative effects on the major agronomic traits such as plant height, seed setting and 1000 grain weight were observed in *OsPUTs* triple mutant plants (Fig. 1G), suggesting that manipulation of *OsPUT1/2/3* genes could be used to generate paraquat-resistant rice plants in field practice.

As a fast acting and non-selective herbicide for green plant tissue, paraquat significantly causes chloroplast damage. Fully expanded leaves were emerged in buffers containing 3 mM MES, 0.1% Tween-20 and series of concentrations of paraquat for 2 days. Although paraquat treatment also decreased chlorophyll content in the triple mutant, the WT plants showed higher paraquat sensitivity than the *OsPUTs* triple mutant plants in terms of leaf color (Fig. 1H). Under electron microscopy, mesophyll chloroplasts in *OsPUTs* mutant plants with paraquat treatments were regularly arranged, which was similar to that in non-treated plants. In contrast, round and swollen chloroplasts with disorganized lamellae were observed in paraquat-treated WT plants (Fig. 1I). Further HPLC–MS/MS analysis confirmed that paraquat absorption was dramatically reduced in *OsPUTs* mutant plants comparing to that in WT plants in both leaves and roots (Fig. 1J–K), which is consistent with the observed phenotypes and the function of *OsPUTs* in paraquat uptake in rice.

CRISPR/Cas-based genome editing has emerged as one of the most advanced systems for engineering crop genomes techniques, and it has been successfully employed to develop transgene-free crops with desired traits in recent years (Gao 2021). Under conditions of labor or resource scarcity, direct seeding, rather than transplantation, is the preferred mode of rice cultivation. However, rice seedlings are vulnerable to weeds competition and a clean seedbed is required, which could be achieved by killing weeds with paraquat. OsPUT1/2/3 were previously shown to transport polyamines and paraquat in yeast cells (Mulangi et al. 2012a, b). Our current results have demonstrated that genetic editing of *OsPUTs* could be useful for producing paraquat-resistant rice plants, since the *OsPUTs* triple mutant plants were tolerant to paraquat with the spray concentration up to 200 μM, a concentration higher than that used by farmers (approximately 140 μM) (Alhag et al. 2021; Li et al. 2013). Previously, loss-of-function of *AtPAR1* and knock-down expression of *OsPAR1/OsPTU3* has been shown to increase paraquat resistance in *Arabidopsis* and rice, respectively (Li et al. 2013). Our results showed the application potential to create paraquat-resistant transgenic-free rice plants. Since the PUT proteins are very conserved across different plant species (Alhag et al. 2021; Mulangi et al. 2012b), precise editing of *PUT* genes is also promising for engineering other crops for weed control.

## Conclusion

Together, our results show that these three *OsPUTs* loci are ideal targets for blocking paraquat uptake in rice, and CRISPR/Cas9-targeted mutagenesis of these loci provides new strategies to generate paraquat resistance rice varieties without obvious yield penalty.

## Supplementary Information

Below is the link to the electronic supplementary material.

Supplementary file1 (DOCX 1870 KB)

## Data Availability

All data generated or analysed during this study are included in this published article and its supplementary information files.
