# Supplementary material for: Disruption of three polyamine uptake transporter genes in rice by CRISPR/Cas9 gene editing confers tolerance to herbicide paraquat
Source: aBIOTECH. 2022 Jun 25;3(2):140–5. doi: 10.1007/s42994-022-00075-4 (PMC9590464; doi:10.1007/s42994-022-00075-4)
Supplement: Supplementary file 1 — Supplementary file1 (DOCX 1870 KB) [file 42994_2022_75_MOESM1_ESM.docx]

**Table S1 Primers used in vector construction**

|  | Target | Promoter | Primers |
| --- | --- | --- | --- |
| OsPUT1 | AGGACAGCGTGGGCGCGGCC | OsU3 | gRT1:GGACAGCGTGGGCGCGGCCGTTTTAGAGCTAGAAAT |
|  |  |  | OsU3T1:GGCCGCGCCCACGCTGTCCTGCCACGGATCATCTGC |
| OsPUT2 | GTAGAAGATGAGCGCGACGA | OsU6a | gRT2:TAGAAGATGAGCGCGACGAGTTTTAGAGCTAGAAAT |
|  |  |  | OsU6aT2:TCGTCGCGCTCATCTTCTACGGCAGCCAAGCCAGCA |
| OsPUT3 | CTCAGCCTGTCTTCCAATGG | OsU6b | gRT3:CTCAGCCTGTCTTCCAATGGGTTTTAGAGCTAGAAAT |
|  |  |  | OsU6bT3:CCATTGGAAGACAGGCTGAGCAACACAAGCGGCAGC |

**Table S2 Potential off-target sites analysis**

| Gene | Potential off-target sites | Region | OsPUTs-KO-1 | OsPUTs-KO-2 |
| --- | --- | --- | --- | --- |
| *OsPUT1* | site 1: AAGTCCGGCGTCCCGGCGCTCGG | Intergenic | No mutagenesis | No mutagenesis |
|  | site 2: GAGATCGGCGTCCCGGCGCTAGG | Os07g0489300/exon | No mutagenesis | No mutagenesis |
|  | site 3: GAGCTCGGCGTCCCGGCGCTCAG | Os07g0489200/exon | No mutagenesis | No mutagenesis |
| *OsPUT2* | site 1: GAAGAAGATGAGCGCGACGGTGG | Os06g0572400/exon | No mutagenesis | No mutagenesis |
|  | site 2: GAAGAAGAGGAGCGCGACGAGAG | Os06g0273700/exon | No mutagenesis | No mutagenesis |
|  | site 3: CTTGTAGTTGAGCGCGACGAGGG | Intergenic | No mutagenesis | No mutagenesis |
| *OsPUT3* | site 1: TTCATCCAGTTTTCCAATGGAAG | Intergenic | No mutagenesis | No mutagenesis |
|  | site 2: CTCAGCCTCTCCTCCAATGCTGG | Os10g0198600/intron | No mutagenesis | No mutagenesis |
|  | site 3: CTCATCCTGTCTTCCAATAACAG | Os05g0187100/exon | No mutagenesis | No mutagenesis |
